# Quasiparticle Self-Consistent *GW*-Bethe–Salpeter Equation Calculations for Large Chromophoric Systems

**DOI:** 10.1021/acs.jctc.2c00531

**Published:** 2022-10-06

**Authors:** Arno Förster, Lucas Visscher

**Affiliations:** Theoretical Chemistry, Vrije Universiteit, De Boelelaan 1083, NL-1081 HVAmsterdam, The Netherlands

## Abstract

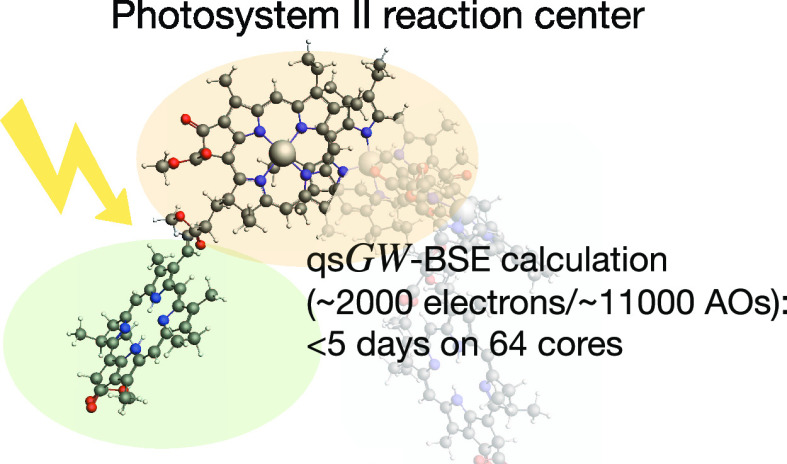

The *GW*-Bethe–Salpeter equation (BSE) method is promising for calculating the low-lying excitonic states of molecular systems. However, so far it has only been applied to rather small molecules and in the commonly implemented diagonal approximations to the electronic self-energy, it depends on a mean-field starting point. We describe here an implementation of the self-consistent and starting-point-independent quasiparticle self-consistent (qs*GW*)-BSE approach, which is suitable for calculations on large molecules. We herein show that eigenvalue-only self-consistency can lead to an unfaithful description of some excitonic states for chlorophyll dimers while the qs*GW*-BSE vertical excitation energies (VEEs) are in excellent agreement with spectroscopic experiments for chlorophyll monomers and dimers measured in the gas phase. Furthermore, VEEs from time-dependent density functional theory calculations tend to disagree with experimental values and using different range-separated hybrid (RSH) kernels does change the VEEs by up to 0.5 eV. We use the new qs*GW*-BSE implementation to calculate the lowest excitation energies of the six chromophores of the photosystem II (PSII) reaction center (RC) with nearly 2000 correlated electrons. Using more than 11,000 (6000) basis functions, the calculation could be completed in less than 5 (2) days on a single modern compute node. In agreement with previous TD-DFT calculations using RSH kernels on models that also do not include environmental effects, our qs*GW*-BSE calculations only yield states with local characters in the low-energy spectrum of the hexameric complex. Earlier works with RSH kernels have demonstrated that the protein environment facilitates the experimentally observed interchromophoric charge transfer. Therefore, future research will need to combine correlation effects beyond TD-DFT with an explicit treatment of environmental electrostatics.

## 1 Introduction

**Figure 1:**
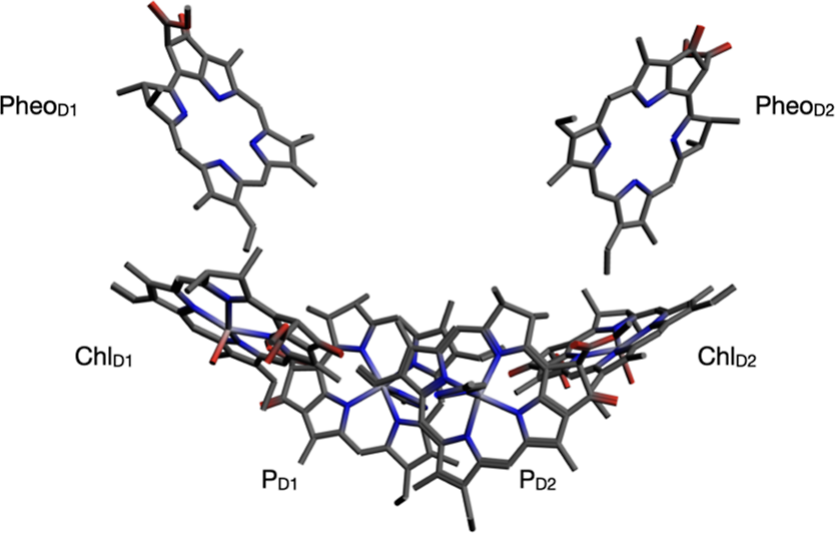
Chromophores of the photosystem II reaction center.

The absorption of photons by a molecule or a material upon interactions with electric radiation is a key process in the conversion of light into chemical or electrical energy. In the photosystem II (PSII) reaction center (RC), photons are captured by chromophoric complexes, which then leads to the generation of free charge carriers.^1^ In the first step of this process an electron–hole pair is formed, where electron and hole are bound due to their Coulombic interaction.^2^ Such bound electron–hole states are commonly referred to as excitons and correspond to the energies of the absorbed photons.^3^ In the current work, we look at the characterization of such low-lying excited states of the RC of PSII, which is at the heart of photosynthetic function.^4^ As shown in Figure 1, the PSII RC contains six chromophores, a “special pair”,^5,6^ of two chlorophyll *a* (chla) molecules (*P*_D1_ and *P*_D2_), flanked by two more chla (Chl_D1_ and Chl_D2_), and two pheophytin *a* (Pheo_D1_ and Pheo_D2_) molecules, with around 2000 electrons in total. By now, it has been firmly established that the primary events of charge separation in PSII are determined by a complex interplay of all these six chromophores.^7^ Therefore, all six chromophores should ideally be treated on a quantum mechanical level and their couplings need to be taken into account.

In most current calculations of larger biomolecular complexes, one resorts to Hartree–Fock (HF)^8,9^ or time-dependent (TD) density functional theory (DFT) with a range-separated hybrid (RSH) exchange–correlation kernel.^7,10−16^ RSHs frequently offer good agreement with the experiment for Chla monomers and dimers,^13,17,18^ but large deviations with respect to more advanced multi-configurational-^18,19^ and wave function-based methods have also been observed.^20^ To mitigate such errors, RSHs can be parametrized empirically for each system under investigation (as done in refs (21) and (22)), but this makes them non-transferable and unreliable for general applications. Systematic tuning procedures for range-separated functionals have been suggested as well.^23−26^ Those however always require to perform exploratory calculations to find the ideal range–separation parameter. Furthermore, heterogeneous systems like multi-chromophoric complexes might require different range separation parameters for different regions of the complex.^27^

Turning to wave function-based methods for excited states, we find the second-order algebraic diagrammatic construction scheme [ADC(2)]^28,29^ and coupled cluster^30−34^ with approximate doubles (CC2)^35^ easy to apply and reasonably cost-efficient. CC2 results are typically in good agreement with more involved methods like equation-of-motion (EOM) CC^36^ with singles and doubles (EOM-CCSD) or similarity-transformed (ST) EOM^37,38^-CCSD.^39,40^ For these methods, we are aware of one study of a tetrameric model by Suomivuori et al.^41^ using ADC(2) together with the spin-opposite-scaled^42^ and reduced-virtual-space (RVS)^43^ approximations. Unfortunately, they did not include the pheophytin chromophores in their calculations, which are known to play a key role in the initial charge separation immediately after photoexcitation.^14,44−46^ This is potentially possible, but we note that most applications of wave function-based methods^18,20,47,48^ focus on single chromophores. Utilizing subsystem methods,^49−55^ the applicability of these methods can be extended. In this family of methods, one describes the full RC by an effective Hamiltonian with a limited amount of levels for each chromophore. The information needed to build such an effective Hamiltonian are the monomeric excitation energies as well as the inter-monomeric couplings. These parameters can be computed in a first-principles manner with various electronic structure methods.^56−58^ While the subsystem approach can be used with high-level monomer calculations, a drawback is that commonly used approximations to calculate the couplings between the chromophores are often not accurate enough.^17,43,59^ In the current work, we will therefore examine how large a system can be treated directly without having to resort to partitioning and subsystem methods. As the states of interest are the lowest energy ones, we thereby focus on a limited number of states but describe them in a supermolecular fashion that fully accounts for all intermolecular couplings of the chromophores.

Our approach is based on the *GW*-BSE method that we will briefly summarize in the following. We first note that energy levels of the excitonic states correspond to the poles of the 2-particle generalized susceptibility.^60−62^ This quantity can be obtained from the interacting single-particle Green’s function *G*_1_ and the electronic self-energy Σ, a non-local, non-Hermitian, and frequency-dependent one-electron operator, via a Bethe–Salpeter equation (BSE).^63−65^*G*_1_ is obtained from a Dyson equation with Σ as its kernel, while Σ itself depends implicitly on the 2-particle Green’s function.^65−67^ As obtaining the full generalized susceptibility requires *N*^6^ operations, it is advantageous to decouple the BSE from the Dyson equation for *G*_1_. This is done by using an approximation to the self-energy, which only depends on the density–density response.^68,69^ A popular example is the *GW* approximation (GWA), with the screened Coulomb interaction *W*(70,71) calculated within the random phase approximation (RPA).^72^ Typically, the Dyson equation for *G*_1_ is solved within the GWA first. Only afterward, the non-interacting 2-particle Green’s function and the corresponding kernel in its zero-frequency limit are constructed and one solves for a few or all roots of the generalized susceptibility.^73−75^ If only a few excitonic states are needed, one may thereby use computationally efficient iterative diagonalization techniques.^75,76^ This procedure is known as the *GW*-BSE method and is increasingly applied to compute the lowest electronically excited states of molecular systems.^55,58,77−106^

For such applications, the *GW* part is typically the computational bottleneck of a *GW*-BSE calculation.^90,92,104^ The issue has been addressed over the last years: many implementations of *G*_0_*W*_0_ and ev*GW* with reduced asymptotic scaling with system size have been developed^107−117^ often producing results in excellent agreement with conventional *GW* implementations.^107,111,112^ Another issue is related to the common approximations in solving the *GW* equations. Typical calculations start from a Kohn–Sham (KS)-DFT or HF Green’s function followed by a perturbative update of the QP energies (*G*_0_*W*_0_).^118,119^ This procedure comes with the notable disadvantage that the outcome of such a calculation will heavily depend on the choice of the underlying exchange–correlation (XC) functional.^84,120−123^ Achieving self-consistency in the eigenvalues only (ev*GW*) can remove this dependence on the initial density functional approximation to a large extent but not completely.^90,104,124^

Instead, one can also start from the full *GW* self-energy and take the Hermitian part only to arrive at a set of effective single-particle equations.^125,126^ In QP self-consistent *GW* (qs*GW*), then only the low-frequency limit of the self-energy is considered,^127−129^ and the non-interacting *G*_1_ closest to the *GW G*_1_ is selected.^130^ While this approach has been shown to be more accurate than *G*_0_*W*_0_ and ev*GW* for a wide range of molecular systems,^131^ qs*GW* has until now rarely been used in molecular calculations. With only a few exceptions,^132,133^ low-order scaling *GW* algorithms only target the screened Coulomb interaction. This is a reasonable choice if one only wishes to calculate the diagonal elements of the self-energy. The computational cost for obtaining the full self-energy is however much larger, and most implementations therefore become inefficient if the full self-energy is required. To address this issue, we have recently presented a low-order scaling implementation of qs*GW*.^133^ In the present work, we combine it with an efficient solver for the BSE, resulting in a fast, low-scaling, and starting-point independent implementation of the GW-BSE approach.

The *GW*-BSE method has recently been shown to reproduce experimental low-lying excitation energies of Chls with high accuracy.^104,134^ So far, it has only been applied to monomeric models of PSII. In this work, we will first give a brief account of the (low-scaling) implementation of the *GW*-BSE approach in Section 2. After describing some technical details of our calculations in Section 3, in Section 4 we first contrast qs*GW*-BSE to ev*GW*-BSE for single chromophores and chromophore dimers and confirm the excellent agreement of the former with experimental data. We then use the qs*GW*-BSE implementation to calculate the low-lying excitation of the hexameric complex with 2000 correlated electrons in total. Finally, Section 5 summarizes and concludes this work.

## 2 Theory

### 2.1 *GW*-BSE Formalism

The interacting *n*-particle Green’s functions corresponding to an *N*-electron system with the ground state Ψ_0_^(*N*)^ are defined by
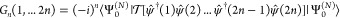
1Here, *T* is the time-ordering operator, ψ̂ is the field operator, and a number 1 = (***r***_1_, σ_1_, *t*_1_) collects space, spin-, and time indices. The relevant cases are *n* = 1, 2. For the *n* = 2 case, we further restrict ourselves to the excitonic part only with *t*_3_ = *t*_4_ and *t*_1_ = *t*_2_.

The single-particle Green’s function can be related to its non-interacting counterpart *G*_1_^(0)^ by a Dyson equation
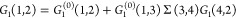
2in which the self-energy operator Σ appears.^135^ In (2) and in the following, integration over repeated indices is implied. The reduced 2-particle Green’s function
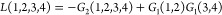
3fulfills a BSE,^62,136^
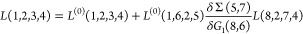
4where^136^
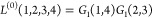
5

The local Hartree kernel is obtained by approximating Σ with the Hartree potential
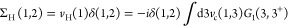
6where *v*_c_ is the Coulomb potential and . Calculating
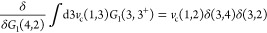
7and inserting the result into (4) one then obtains
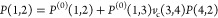
8with
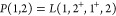
9being the *v*_c_-reducible density–density response function in the RPA and
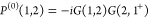
10*P* is related to the screened Coulomb interaction *W* by^70^
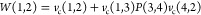
11which can be used to define the *GW* self-energy
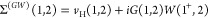
12

Equations 2, 8, and 10–12 constitute a self-consistent set of equations, usually referred to as the *GW*-approximation.

By splitting the self-energy into Hermitian and anti-Hermitian part and discarding the latter one, we can restrict the solution of (2) to its QP part only.^125,126,137,138^ We then have an effective single-particle problem and restricting the self-energy further to its static limit and transforming into the molecular orbital basis  (in which the single-particle Hamiltonian is diagonal), we arrive at
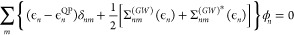
13where the ϵ_*n*_ are the single-particle energies. Solving eqs 8 and 10–13 self-consistently is known as the qs*GW* approximation within the RPA.^127−129^

After solving the qs*GW* equations self-consistently, we can then use the zero-frequency limit of the self-energy (12) for the kernel of (4). As it is typically done, we also set δ*W*/δ*G* ≈ 0. This is referred to as the qs*GW*-BSE approach. After Laplace transformation to the complex frequency plane, eq 4 can be transformed into an eigenproblem in a basis of particle-hole states whose solution provides the Lehmann representation of *L* (see refs (139) or (140) for detailed derivations)
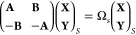
14Ω_*S*_ is a neutral excitation energy, (**X**,**Y**)_*S*_^*T*^ contains the expansion coefficients of the corresponding eigenvector and for a closed-shell system the matrix elements of **A** and **B** are, respectively, defined as
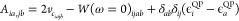

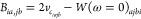
15where we have chosen to reserve the labels *i*, *j*,... for occupied and *a*, *b*,... for virtual orbitals. The QP energies entering the equations are the ones from (13).

### 2.2 Implementation

For our implementation of the qs*GW* methods, we refer to our previous work.^110,133,141^ We expand single-particle Green’s functions and the self-energy in a basis of Slater type functions (primary basis) which is related to the MOs by
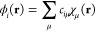
16while all quantities appearing in (11) are expanded in a basis of auxiliary fit functions (auxiliary basis). We then switch to the particle-hole basis to solve (14), whereby the matrix elements in (15) are expanded in the basis of MOs.

Because we do not use the screened interaction at zero frequency in our *GW* implementation, we calculate the zero-frequency component of *P* from the imaginary time representation of the polarizability by
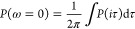
17and we then use (11) to obtain *W*(ω = 0).

Replacing the matrix elements of the screened Coulomb interaction by the bare ones in (15), and using the HF self-energy in (13), the TD-HF method is obtained. It is clear that any solver which can be used to solve (14) in the TD-HF case can also be used for *GW*-BSE. We use an extension of the Davidson algorithm^142^ originally proposed by Stratmann and Scuseria.^76^ It solves (4) by projecting the generalized problem
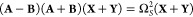
18on a sequence of orthonormal subspaces
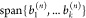
19in which (18) is solved. *k* denotes the size of the *n*th subspace and the *b*_*k*_ are linear combinations of particle-hole states. The vectors forming the subspace are then updated until the subspaces are converged. The procedure can be interpreted as an iterative optimization of the basis of particle-hole states, where the part which does not carry useful information (i.e., the particle-hole transitions which do not contribute to the low-lying excitons) is projected out.

The time-determining step in the diagonalization is the projection of the eigenproblem in the full space on the subspaces. The term containing the bare Coulomb potential is easily evaluated following the procedure in ref (143). For the matrix elements of the screened interaction in the (*n* + 1)th subspace iteration, we define a column in the subspace labeled by *s*_*i*_, *s*_*j*_,..., *s*_*a*_, *s*_*b*_,..., respectively, as
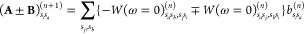
20

In the minus case, this is equivalent to the evaluation of the greater or lesser component of self-energy for a single imaginary time point. In the plus case, a similar algorithm can be used, but the resulting matrix needs to be antisymmetrized. We solve (20) in the basis of Slater functions and then transform to the basis which spans the subspace. For detailed working equations, we refer to Appendix B.

A key element in our approach is to use pair-atomic density fitting (PADF)^110,144−148^ to calculate the transformation from auxiliary basis to primary basis and back. In PADF, all the coefficients in the transformation matrix corresponding to auxiliary functions, which are not centered on the same atoms as the primary basis functions are restricted to zero. While making the resulting basis transformation very efficient this also is an approximation which does not necessarily conserve important properties of the original matrices, like, for example, positive definiteness of the Coulomb potential.^147^ These deficiencies can always be traced back to products of diffuse Slater functions, which are difficult to expand in the auxiliary basis. To overcome these issues, we introduce a projection technique to remove problematic linear dependencies from the matrices appearing in eq 20. This projection technique is described in Appendix C.

## 3 Computational Details

All calculations have been performed with a locally modified development version of ADF2022.1^149,150^ The *GW* implementation is the same as outlined in refs (110)(133)(141), except for the modification outlined in Appendix C.

For the hexameric unit of PSII, we used the structure of ref (16), which has therein been optimized at the PBE level of theory taking into account environmental effects using a QM/MM approach. Dimer structures have been optimized in this work using CAM-B3LYP-D3(BJ), a triple-ζ + polarization (TZP)^151^ basis set, and *Good* numerical quality. The monomer structures used in Sections 4.1 and 4.2 are taken from the structure published in ref (12) based on the experimental structure at 1.9 Å resolution by Umena et al.^152^ and where the positions of the hydrogen atoms have been optimized using a semi-empirical model with all other coordinates frozen. All structures used in this work can be found in the Supporting Information.

We also benchmarked the basis set dependence of the *GW*-BSE calculations using the larger TZ3P and QZ6P basis sets^141^ for Chla monomers in Section 4.2. All qs*GW*-BSE results shown there have been obtained with the *veryGood* auxiliary basis. This allows us to reliably compare excitation energies obtained with different primary basis sets. TZ3P and QZ6P contain *f*-functions for second-row atoms and for such basis sets, the *Good* auxiliary fit set is generally insufficient. For monomers, we calculate the lowest 6 eigenstates of (18).

For chromophore dimers, we calculated the lowest 6 eigenstates of (18), using TZP (triple-ζ + polarization)^151^ as the primary basis set, *Good* numerical quality, and 16 imaginary time and frequency points each. In all calculations for monomers and dimers, we terminate the sequence of subspace iterations if all eigenvalues are converged within 10^–5^ Ha (0.27 meV).

In the *GW*-BSE calculations of the excited states of the hexamer, we used the TZP basis set, *Basic* numerical quality, and 12 imaginary time and frequency points each. We restrict the basis, in which we solve the BSE to the subspace spanned by all particle-hole pairs with transition energies below 1.5 Ha. In agreement with earlier *GW*-BSE studies for such systems,^80^ we found this approximation to change the low-lying excitation energies by only around 10–20 meV compared to calculations including all particle-hole pairs.^153^ This improves numerical stability of our algorithm and accelerates the convergence of the subspace iterations in the Davidson algorithm. We perform 8 subspace iterations in the Davidson algorithm and calculate the 24 lowest eigenstates of (18). This is sufficient to converge the low-lying excited states to within less than 5 meV. We also calculated the low-lying excited states of the same system using TD-DFT with the ωB97-X kernel using the same numerical settings. However, in contrast to our *GW*-BSE calculations, we calculated the 12 lowest states and converged all eigenvalues to within 10^–6^ Ha.

In all calculations, we took into account scalar relativistic effects in the zeroth-order regular approximation.^154−156^ The threshold ϵ_*s*_ described in Appendix C has been set to 5 × 10^–3^. Also, in all KS calculations, we set the threshold for the Löwdin orthogonalization to 5 × 10^–3^. If not stated otherwise, in all qs*GW* calculations, we first perform a PBE0 calculation with 40% exact exchange (PBEH40), which is a good preconditioner for qs*GW* and leads to fast convergence.^157^ Aside from numerical inaccuracies, the final results are independent of this choice, which we have verified in ref (133) and which we will verify also for the case of Chla in the next section. For qs*GW*, we terminate the calculations when the Frobenius norm of the difference between the density matrices of two subsequent iterations falls below 5 × 10^–9^.^133^ We also performed ev*GW*-BSE calculations based on the LDA and PBEH40 functionals (ev*GW@*LDA, ev*GW@*PBEH40). We terminate the ev*GW* calculations if the HOMO QP energy difference between two subsequent iterations falls below 3 meV.

To compare our method to the RSH TD-DFT approach, we also performed calculations using the CAMY-B3LYP and ωB97-X kernels using the TZP basis set and *Good* numerical quality. We also calculated the electrochromatic shifts due to the presence of the protein environment using the conductor-like screening model (COSMO),^158−160^ as implemented in ADF.^161^ these results are shown in Appendix A. Following ref (41), we set the dielectric constant of the environment to a value of 4.0 in these calculations, which should approximately account for solvent and protein environments.

## 4 Results

### 4.1 Starting-Point Dependence of *GW*-BSE Calculations

**Figure 2:**
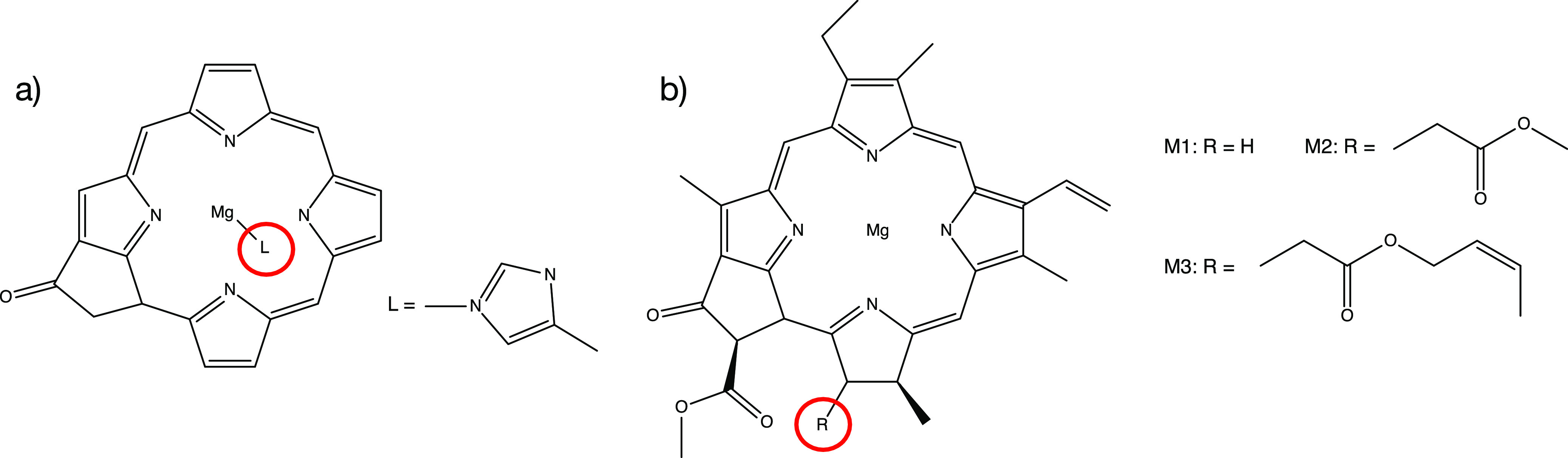
Different models of Chla used in this work: (a) model used by Suomivuori et al.^41^ with ligating histidine residue. (b) Models without histidine residue but containing all ligands at the chlorin core and different models for the phytyl chain (M1, M2, and M3, respectively).

As discussed in the introduction, its starting point independence is a major advantage of qs*GW* over ev*GW*. To verify the starting point independence of our implementation, we report here vertical excitation energies (VEEs) for qs*GW* and ev*GW* for the M2 model in Figure 2b with 82 atoms in total for the LDA, PBE, PBEH40, and HF starting points. We thereby use a tighter convergence criterion of 1 meV for the HOMO QP energy for ev*GW* than the default value. The results for the *Q*_*y*_ excitation are shown in Table 1. The qs*GW* calculations converge to the same HOMO–LUMO gap within an accuracy of 10 meV within less than 10 iterations. This also results in *Q*_*y*_ excitation energies, which are converged within 10 meV. The remaining differences are due to numerical noise in the imaginary frequency and time grids used in the *GW* calculations, which then translates into uncertainties in the analytical continuation of the self-energy to the complex plane.^111,141^ The differences in the HOMO–LUMO gaps of the ev*GW* calculations are much larger and differ by almost 300 meV between ev*GW@*LDA and ev*GW@*HF, which results in *Q*_*y*_ excitation energies differing by about 80 meV. This is the most extreme case, for starting points other than HF there are only very small differences between the different ev*GW* results. This has already been observed in ref (104). Because the computational overhead of a qs*GW* calculation is negligible compared to ev*GW* (5.79 vs 5.67 core hours per iteration) and the number of iterations needed for convergence is essentially the same, there is little advantage to be gained by using ev*GW* instead of the more robust qs*GW* approach.

**Table 1:** HOMO–LUMO Gap, Value of the *Q*_*y*_ Excitation for Different Starting Points, Number of Iterations Until Convergence and Time per *GW* Iteration, Measured in Core Hours, for qs*GW* and ev*GW*^a^

	ev*GW*				qs*GW*			
	gap	*Q*_*y*_ [eV]	*n*_*I*_	*t* [h]	gap	*Q*_*y*_ [eV]	*n*_*I*_	*t* [h]
LDA	4.405	1.764	9	5.67	4.499	1.752	9	5.79
PBE	4.417	1.837	9		4.501	1.745	10	
PBEH40	4.476	1.772	7		4.493	1.760	8	
HF	4.671	1.766	9		4.496	1.753	9	

a Calculations were performed on a 2.2 GHz intel Xeon (E5-2650 v4) node (broadwell architecture) with 24 cores and 128 GB RAM.

### 4.2 Basis Set Errors

Next, we investigate the dependence of the *Q*_*y*_ excitation energy on the basis set size. For *GW* calculations, it is well known that individual QP energies converge slowly with respect of the size of the single-particle basis. In practice, extrapolation techniques are needed to obtain converged results.^162−164^ For QP energy differences, which are entering the BSE, the situation is much better because the basis set error for the QP energies usually have the same sign.^163^ In Table 2, we compare the lowest excitation energies calculated with different basis sets for the two different Chla models M1 and M2 shown in Figure 2b). For ev*GW* and qs*GW*, the QZ6P VEEs are only slightly lower than the TZP ones, indicating that they are almost converged also with the smaller basis set. These errors are certainly smaller than other possible sources of error in our calculations like shortcomings of *GW*-BSE or uncertainties in structural parameters. Therefore, to a very good approximation, we can ignore the basis set incompleteness error in all of the following TZP calculations.

**Table 2:** VEEs for M1 and M2 with Different Basis Sets for qs*GW*-BSE and ev*GW@*LDA-BSE^a^

	ev*GW*@LDA-BSE						qs*GW*-BSE					
	M1			M2			M1			M2		
	*Q*_*y*_	*Q*_*x*_	*B*	*Q*_*y*_	*Q*_*x*_	*B*	*Q*_*y*_	*Q*_*x*_	*B*	*Q*_*y*_	*Q*_*x*_	*B*
TZP	1.74	1.93	2.68	1.76	1.94	2.71	1.72	1.98	2.84	1.74	2.00	2.86
TZ3P	1.77	1.96	2.72	1.79	1.98	2.76	1.72	1.98	2.84	1.73	1.97	2.84
QZ6P	1.71	1.94	2.64	1.74	1.92	2.68	1.71	1.96	2.80	1.71	1.96	2.84
Δ_*TQ*_	0.03	–0.01	0.04	0.02	0.02	0.03	0.01	0.02	0.04	0.03	0.04	0.02

a The values in the last row denote the differences in VEEs calculated with the TZP^151^ and QZ6P^141^ basis sets. All values are in eV.

### 4.3 Comparison to Experiments and Different Ab-initio Calculations

#### 4.3.1 Monomers

Next, we assess the accuracy of qs*GW*-BSE by comparison to experimental gas-phase data for Chla by Gruber et al.^165^ In Table 3, we directly compare VEEs calculated with different computational methods to the experimental VEE, which has recently been extracted from the experimental spectrum by Sirohiwal et al.^47^ The domain based local pair-natural orbital^166,167^ (DLPNO)-STEOM-CCSD^168−170^ results are taken from ref (47), while the ev*GW@*LDA-BSE/6-311++G(2d,2p) results calculated using MOLGW^171^ are by Hashemi and Leppert.^104^ Two different, gas-phase optimized structures have been used: one has been optimized at the CAM-B3LYP-D3(BJ)/def2-TZVP level of theory by Sirohival et al.,^47^ while the other has been optimized by Hashemi and Leppert using B3LYP/def2-TZVP.

**Table 3:** VEEs for Chla Calculated with Different Quantum Chemical Methods for Two Different Gas-Phase-Optimized Structures and Experimental Reference Data^a^

	*Q*_*y*_	*Q*_*x*_	*B*	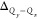
exp. (VEE)	1.99	2.30	3.12	0.31
exp. (band max)	1.94	2.23	3.08	0.29
CAM-B3LYP-D3(BJ)/def2-TZVP Optimized Structure				
DLPNO-STEOM-CCSD	1.75	2.24	3.17	0.49
qs*GW*	1.97	2.29	3.15	0.32
ev*GW@*PBEH40	1.98	2.29	3.15	0.31
ev*GW@*LDA	1.94	2.20	3.01	0.26
CAMY-B3LYP	1.94	2.23	3.08	0.29
ωB97-X	2.10	2.71	3.57	0.61
B3LYP/def2-TZVP Optimized Structure				
ev*GW*@LDA-BSE(ADF/TZP)	1.85	2.09	2.91	0.24
ev*GW*@LDA-BSE(MOLGW/6-311++G(2d,2p))	1.85	2.13	2.91	0.28

a All values are in eV.

We performed ev*GW@*LDA-BSE calculations for both structures. Our results for the CAM-B3LYP-D3(BJ)-optimized structure are consistently around 0.1 eV lower than the ones for the B3LYP optimized structure. This illustrates the large influence of small changes in structural parameters on the final excitation energies. However, CAM-B3LYP has been shown to describe the structural features of chlorophyll monomers very well.^47,172^ For the B3LYP optimized structure, we can compare our herein calculated VEEs to the ones from Hashemi and Leppert calculated on the same level of theory. Except for the *Q*_*x*_ excitation energies, which are slightly different (40 meV), we find a perfect agreement between both implementations.

All ev*GW* results agree very well with qs*GW* also for Chla. All *GW*-BSE results for the CAM-B3LYP-D3(BJ) optimized structure are in excellent agreement with the experimental values. For instance, the qs*GW*-BSE VEEs agree all with the experimental VEEs within 30 meV. On the other hand, DLPNO-STEOM-CCSD not only severely underestimates the *Q*_*y*_ excitation energy, but it also overestimates the gap between both *Q*-bands, , considerably. Considering this difference, we note that STEOM-CCSD is not necessarily a reliable reference for qs*GW*. In STEOM-CCSD, a much larger number of diagrams is considered in the single- and two-particle Green’s functions compared to *GW*.^173^ QP approximations to *GW* approximate the effect of these diagrams instead by neglecting the vertex.^129^ The diagrams contained in *GW* are not a subset of the ones contained in EOM-CCSD but only of those contained in EOM-CCSDT.^173^ Accounting for excitations to triples (at least to some extent) is known to be of high importance for the reliable description of charged^174^ and neutral excitations.^39,40,175^ Consequently, STEOM-CCSD shows mean signed errors compared to EOM-CCSDT calculations of around 0.1 eV for a set of medium organic molecules, but errors can be as large as 0.5 eV in some cases.^39^ Moreover, apart from the neglect to triple excitations, the DLPNO approximation can also introduce some artifacts. The pairs, which are treated on the CC level, are selected based on an MP2 calculation,^167^ which is not always reliable for systems with strongly screened electron–electron interactions.^176,177^

Lastly, the RSH kernels CAMY-B3LYP and ωB97-X, which are typically used in computational studies of the PSII RC^11−13,16^ give very different results. CAMY-B3LYP is actually in excellent agreement with the experiment and the *GW*-BSE calculations, while ωB97-X gives much too high excitation energies and also massively overestimates .

#### 4.3.2 Dimers

In Table 4, we show the low-lying excitations of *GW*-BSE calculations for different models of *P*_D1_–*P*_D2_. The first dimer structure has been optimized in the gas phase by Suomivuori et al. at the B3LYP-D3/def2-SVP level of theory and consists of two Chla monomers, whose structure is shown in Figure 2a. This structure lacks most substituents of the Chlorin core present in Chla (see Figure 2b) which complicates comparison of excitation energies to experimental results. However, these calculations give some indication on the performance of *GW*-BSE in comparison to the RVS-LT-SOS-ADC(2) VEEs by Suomivuori et al. Comparison of experimental band maximum and VEE for a single Chla measured in ref (165) suggests that the VEE of the chlorophyll dimer might be around 1.95 eV (50 meV higher than the band maximum).

**Table 4:** Lowest Six Excitation Energies for Two Different Models of the Chla Dimer^a^

	Ω_1_	Ω_2_	Ω_3_	Ω_4_	Ω_5_	Ω_6_
exp. (VEE)^178^	1.95	(estimated)				
exp. (band max)^178^	1.90					
B3LYP-D3(BJ)/def2-SVP Optimized Structure^b^						
qs*GW*	1.89	1.92	2.07	2.10	2.83	2.92
ev*GW@*PBEH40	1.92	1.95	2.09	2.11	2.84	2.93
ev*GW@*LDA	1.87	1.88	1.90	1.90	2.72	2.75
CAMY-B3LYP	2.12	2.15	2.29	2.32	2.63	2.76
RVS-LT-SOS-ADC(2)^c^	2.04	2.06				
CAM-B3LYP-D3(BJ)/TZP Optimized Structure^d^						
qs*GW*	1.94	1.98	2.25	2.28	2.56	2.68
ev*GW@*PBEH40	1.97	2.02	2.24	2.27	2.58	2.67
ev*GW@*LDA	1.98	1.99	2.16	2.22	2.51	2.64
CAMY-B3LYP	2.12	2.16	2.38	2.43	2.51	2.61
ωB97-X	2.05	2.10	2.63	2.68	3.10	3.27

a All values are in eV.

b The B3LYP-D3(BJ)/def2-SVP structure has been taken from Suomivuori et al.^41^

c Results taken from Suomivuori et al.^41^

d The structure of the M3 dimer has been optimized in this work at CAM-B3LYP-D3(BJ)/TZP.

**Figure 3:**
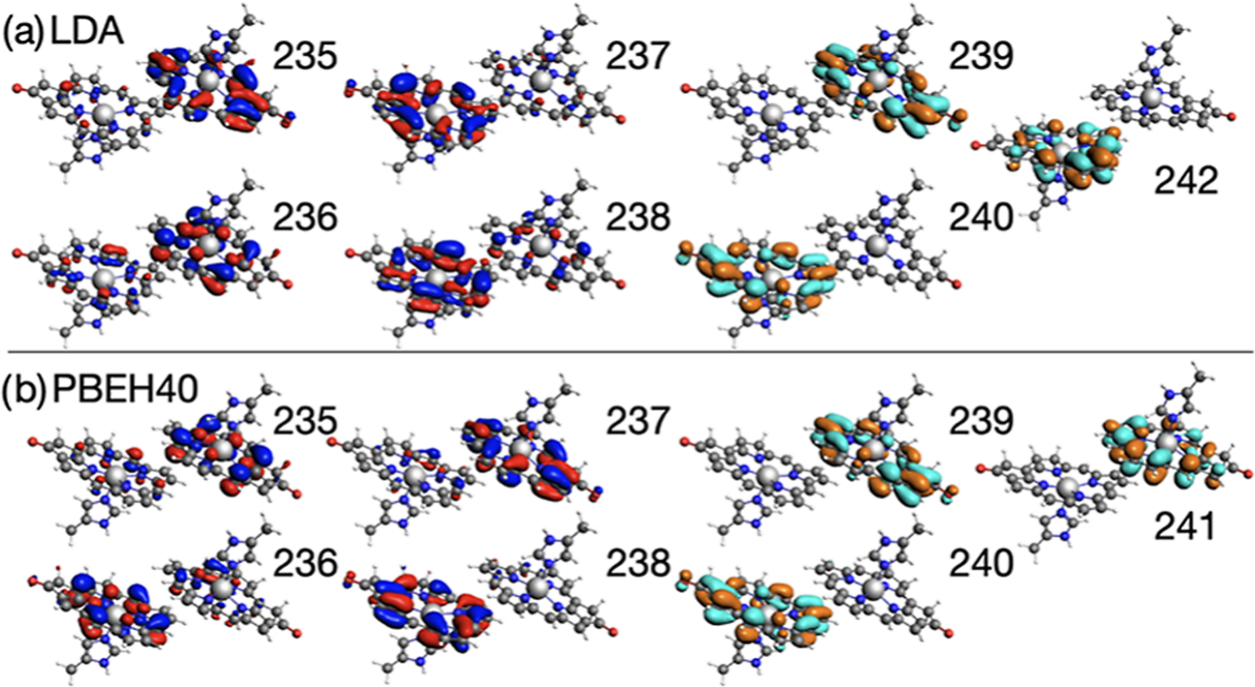
Selected valence single-particle KS orbitals for the Chla dimer (structure by Suomivuori et al.^41^) calculated using LDA and PBEH40.

As for the monomer, the *GW*-BSE results are in excellent agreement with these values, while the RVS-LT-SOS-ADC(2) VEEs are much too high. In contrast to the case of the Chla monomer, CAMY-B3LYP overestimates the VEEs by far. The VEEs, Ω_3_ and Ω_4_, of the BSE calculation based on ev*GW@*LDA are almost 0.2 eV lower than the ones based on ev*GW@*PBEH40 and in the former calculation, the four lowest excited states are almost degenerate. The character of these excitations are compared in more detail in Table 5 with the corresponding KS single-particle orbitals shown in Figure 3. Comparison of the most important contributions to the eigenvector  already shows that ev*GW@*LDA-BSE predicts the lowest excitation to be localized on the *P*_D1_ fragment, while in the ev*GW@*PBEH40-BSE calculation it is delocalized over both monomers with almost equal weights. Using ev*GW@*LDA-BSE, the second excited state has a large contribution of a particle-hole transition located on *P*_D1_, while it is localized on *P*_D2_ using ev*GW@*PBEH40-BSE. Also, the oscillator strengths in Table 5 show that the different excitations differ substantially in their brightness. Together with the large difference in some of the VEEs, this shows that different KS starting points can lead to different excitation characters, even when the eigenvalues are updated self-consistently.

**Table 5:** Characterization and Comparison of the Low-Lying Excited States of Chla Dimer (Structure by Suomivuori et al.^41^) Calculated with ev*GW@*LDA-BSE and ev*GW@*PBEH40-BSE^a^

	ev*GW@*LDA				ev*GW@*PBEH40			
	VEE	character	weight	*f*	VEE	character	weight	*f*
Ω_1_	1.87	238 → 240	0.49	0.08	1.92	238 → 240	0.28	0.30
						237 → 239	0.26	
Ω_2_	1.88	237 → 240	0.22	0.14	1.95	238 → 241	0.41	0.03
		237 → 239	0.17			237 → 239	0.34	
Ω_3_	1.90	236 → 239	0.38	0.13	2.09	235 → 239	0.53	0.04
Ω_4_	1.90	237 → 240	0.37	0.00	2.11	236 → 240	0.49	0.03
		235 → 239	0.31					
Ω_5_	2.72	238 → 239	0.51	0.37	2.84	238 → 239	0.56	0.24
Ω_6_	2.75	237 → 239	0.27	0.14	2.93	237 → 240	0.31	0.20
		237 → 242	0.24					

a Shown are the excitation energies Ω_*S*_ (in eV), the dominant coefficients of the corresponding eigenvector, and the associated particle-hole transitions, as well as the oscillator strengths *f*.

In Table 4, we also show results for a more realistic model of the Chla dimer. Our model consists of two M3 monomers, which includes the first four segments of the phytyl chain in stacked conformation. In Table S1 of the Supporting Information, we show that the final excitation energies are however very insensitive to the particular structural model.

The band maximum of ref (178), which we used as the reference has been measured for a charge tagged dimer. However, as shown in ref (179) for Chla monomers, the final excitation energies are insensitive to the type of charge tag and omitting the charge tag entirely only results in a lowering of the excitation energies of around 30–40 meV.

The excitation energies we report here have been calculated for a geometry optimized at the CAM-B3LYP/TZP level of theory. Excitation energies for geometries optimized with different methods can be found in Table S2 of the Supporting Information. In accordance with ref (47) and our results shown in Table 3, we found the VEEs to be very sensitive to the choice of the functional chosen for geometry optimization. For instance, using PBE-D4/TZP lowers the lowest 2 excitation energies by around 0.1 eV with respect to the CAM-B3LYP-D3(BJ) optimized structure. The data shown in Table S3 in the Supporting Information furthermore demonstrates that VEEs for crystal structures considerably underestimate the experimental values.

The lowest qs*GW*-BSE excitation energy of 1.94 meV is again in an excellent agreement with the VEE of 1.95 eV estimated from the band maximum. As explicitly shown in the Supporting Information and as for the monomers in Table 2, the excitation energies are again rather insensitive to the basis set. Also notice that the remaining small basis set errors will largely cancel with the small error from omitting the charge tag. Again, the lowest two ev*GW*-BSE VEEs are in excellent agreement with the qs*GW*-BSE one and each other, while there are larger differences in higher-lying VEEs. As for the monomer, CAMY-B3LYP massively overestimates the VEEs compared to the experiment.

### 4.4 Six-Chromophore Model of the PSII RC

The most complete model of the PSII RC we consider in this work comprises all six chromophores shown in Figure 1 with 476 atoms in total. Time-resolved spectroscopic experiments^44−46^ show that the primary electron transfer in the RC occurs from an exciton localized on Chl_D1_ to Pheo_D1_, followed by a transfer of the hole to *P*_D1_. This would point to the presence and possible mixing in of low-lying CT states with pronounced Chl_D1_^+^–Pheo_D1_^–^ and *P*_D1_^+^–Pheo_D1_^–^ characters in calculations of excitation energies. In previous TD-DFT calculations using RSH kernels for similar multi-chromophoric models, no low-lying CT state, which could be related to this charge separation pathway have been observed.^11,16^ In recent computational studies, both Sirohiwal et al.^7,16^ and Tamura et al.^14^ demonstrated that the protein environment is crucial for observing the Chl_D1_^+^–Pheo_D1_^–^ CT state at low energies.

The low-lying excitations of the hexameric complex at the qs*GW*-BSE/TZP level of theory are characterized in Table 6. In the Supporting Information, we characterize these excitations in more detail by visualizing the involved single-particle qs*GW* orbitals. We also present results of our own TD-DFT calculations using the ωB97-X kernel as well as for ev*GW*@PBEH40-BSE/TZP. The excitation energies and the oscillator strengths of the six lowest excited states using these different methods are compared in Table 7.

**Table 6:** Lowest qs*GW*@-BSE/TZP Excited States of the Hexameric Chromophore Complex in the RC of PSII^a^

	VEE	*f*	character	weight
Ω_1_	1.89	0.22	*P*_D2_^*^	0.39
			Chl_D2_^*^	0.22
Ω_3_	1.90	0.77	*P*_D2_^*^	0.24
			*P*_D1_^*^	0.14
			Pheo_D2_^*^	0.09
			*P*_D1_^+^–*P*_D2_^–^	0.09
Ω_3_	1.91	0.04	Chl_D1_^*^	0.30
			*P*_D1_^*^	0.24
			Chl_D1_^+^–Pheo_D2_^–^	0.08
Ω_4_	1.92	0.22	Pheo_D2_^*^	0.39
			Chl_D2_^*^	0.16
			Pheo_D2_^*^	0.12
			Chl_D1_^*^	0.09
Ω_5_	1.94	0.01	Chl_D1_^*^	0.23
			Chl_D2_^*^	0.18
			*P*_D1_^*^	0.16
			*P*_D2_^*^	0.15
Ω_6_	1.97	0.20	Pheo_D1_^*^	0.54
			Pheo_D1_^–^–Chl_D1_^+^	0.21
Ω_13_	2.71	0.00	*P*_D2_^+^–Chl_D2_^–^	0.81
			*P*_D1_^+^–Chl_D2_^–^	0.13
Ω_14_	2.73	0.00	*P*_D1_^+^–Chl_D1_^–^	0.70
			*P*_D1_^+^–Pheo_D1_^–^	0.20

a Shown are the excitation energies Ω_*S*_ (in eV), the dominant coefficients of the corresponding eigenvector, and the associated particle-hole transitions, as well as the oscillator strengths *f*.

**Table 7:** VEEs and Oscillator Strengths of the Six Lowest Excited States of the Hexameric Complex at Different Levels of Theory^a^

	qs*GW*-BSE		qs*GW*@PBEH40-BSE		TD-DFT@ωB97-X	
	VEE	*f*	VEE	*f*	VEE	*f*
Ω_1_	1.89	0.22	1.94	0.81	1.92	0.33
Ω_2_	1.90	0.77	1.94	0.32	1.93	0.64
Ω_3_	1.91	0.04	1.96	0.05	1.94	0.14
Ω_4_	1.92	0.22	1.97	0.24	1.96	0.18
Ω_5_	1.94	0.01	1.99	0.15	1.97	0.09
Ω_6_	1.97	0.20	2.00	0.11	1.98	0.07

a All values are in eV.

In agreement with past^11,16^ and our own TD-DFT calculations using the ωB97-X kernel, only states with local characters can be found among the six lowest excitations of the hexamer using both qs*GW*-BSE and ev*GW*@PBEH40-BSE. As shown in Table 7, also the VEEs using the different methods agree within 50 meV. In all methods, the low-lying states are linear combinations of excitonic states involving the frontier orbitals on each chromophore.

At the qs*GW*-BSE level, the two lowest states with pronounced CT characters can be found at 2.7 eV and these cannot directly be linked to charge separation pathways in PSII, which have been observed experimentally.^44−46^ Only the third excited state at the qs*GW*-BSE level of theory at 1.91 eV contains a contribution from a Chl_D1_^+^–Pheo_D1_^–^ particle-hole transition with a small weight, which is entirely absent in our TD-DFT and ev*GW*-BSE calculations. Future studies at the *GW*-BSE level with the inclusion of the environmental electrostatics are needed to rationalize how the Chl_D1_^+^–Pheo_D1_^–^ CT state is influenced by the protein environment at the qs*GW*-BSE level.

### 4.5 Timings

Finally, we briefly comment on the computational effort for different basis sets and methods to calculate the lowest *N*_Ω_ roots of the full hexamer with 476 atoms and 1872 correlated electrons. The computational timings in core hours are given in Table 8. The calculation for the hexamer can be performed in less than 3000 core hours, that is, in less than 2 days on a node with 64 cores. The qs*GW* part of the calculation is slightly cheaper than the BSE part. Notice, that the BSE part of the calculation is roughly as expensive as the TD-DFT calculation with the *W*B97-X kernel if the timings are normalized by the number of states and number of subspace iterations in the Davidson algorithm.

**Table 8:** CPU Times (in Core Hours) to Calculate the *N*_Ω_ Lowest Roots of the Full Hexamer with 476 Atoms and 1872 Correlated Electrons with Different Basis Sets and Methods^a^

				iterations		CPU time		
method	basis	*N*_bas_	*N*_Ω_	qs*GW*	BSE	*GW*	BSE	total
qs*GW*-BSE	TZ3P	11116	12	6	10	3401	3447	7283
	TZP	6256	24	6	8	1074	1729	2924
evGW-BSE	TZP	6256	24	5	8	826	1969	2917
ωB97-X	TZP	6256	12		21		2675	2846

a 39,884 auxiliary basis functions have been used in all calculations. All calculations have been performed on an 2.6 GHz AMD Rome 7H12 node with 64 cores and 16 GB RAM per node.

Low-order scaling implementations like ours which rely on sparsity in the primary basis usually do not scale well with the size of the single particle basis, as can be seen by comparing the timings of the qs*GW*-BSE calculations with different basis sets. We also performed a qs*GW* calculation for the full hexamer with more than 11,000 basis functions using the TZ3P basis set. Here, a single qs*GW* iteration already takes around 540 core hours, which is more than three times longer than one iteration using the TZP basis set. While in this work, the TZP basis set was already sufficient to obtain converged results, typically lager basis sets will be required. Finite basis set correction techniques for many-body perturbation theory might be a promising solution to circumvent this problem.^164,180−182^

For larger calculations, the bottleneck of the computation is the number of auxiliary fit functions *N*_fit_ (almost 40,000 for the hexamer). When large basis sets are used, also large auxiliary fit sets are necessary to guarantee numerical stability in the PADF approach. The same holds true forrelated techniques relying on sparse transformation between matrices in primary and auxiliary bases.^111,112^ For each imaginary time and frequency point, a matrix of size *N*_fit_ × *N*_fit_ ≈ 14 GB needs to be stored for the hexamer. This amounts to storage requirements of almost 500 GB and if we were to double the system size, 2 TB of distributed memory would be needed. In our current implementation, we store these matrices on disk and transfer them to the CPU and back, which becomes very time-consuming.

## 5 Conclusions

So far, applications of the *GW*-BSE method have been limited to rather small molecules.^90,97,104^ We presented here a new implementation of the method, which enables its routine application to much larger systems. As opposed to a recently developed simplified *GW*-BSE scheme,^183^ our implementation does not introduce any empirical approximations to the matrix elements of the BSE Hamiltonian. Our implementation allowed us to calculate the 12 lowest excited states of the complete complex of 6 chromophores in the PSII RC with almost 2000 correlated electrons on the qs*GW*-BSE/TZP level. The calculation with around 6000 primary basis functions could be performed in a little more than 2 days on a single compute node. The corresponding calculation using qs*GW*-BSE/TZ3P with around 11,000 primary basis functions could be performed in around 5 days using the same hardware.

Because the single-particle states are optimized self-consistently, making the results independent of a mean-field reference calculation, qs*GW*-BSE is a theoretically more rigorous approach than ev*GW*-BSE. qs*GW*-BSE calculations for optimized geometries are in excellent agreement with experimental VEEs in the gas phase for Chla monomers and dimers. We have shown here explicitly for Chla dimers that ev*GW*-BSE might lead to different excitations for different starting points. This is in contrast to the generally good agreement for different starting points for monomers^104^ and can be seen as a major shortcoming of ev*GW*-BSE. We therefore conclude that self-consistency in the single-particle states is decisive for a reliable description of the low-lying excitonic states of large chromophoric complexes.

In agreement with previous results and our own calculations on the TD-DFT/RSH level for the full hexameric complex^11^ also ev*GW*-BSE and qs*GW*-BSE only predict states with predominantly local characters in the absence of the protein environment. These states can therefore not be linked to the experimentally observed CT processes.^44−46^ Recent computational studies have established that the environmental electrostatics are responsible for this type of CT.^7,14,16^ Along the lines of previous *GW*-BSE implementations,^91,96,184^ future research needs to focus on ways to explicitly account for the environmental electrostatics in large-scale *GW*-BSE calculations.

## Appendix A

### Electrochromatic Shifts

In this appendix, we quantify the electrochromatic shift of the excitation energies of two monomeric and dimeric as well as one tetrameric model of the PSII RC due to solvent effects and protein environments using a polarizable continuum model. The *Q*_*y*_ excitation energies calculated using CAMY-B3LYP-TD-DFT/TZP with and without implicit solvation are shown in Table 9. Our calculated electrochromatic shifts agree well with experimental values of about 0.12 eV,^20^ which is somewhat surprising and possibly fortuitous because the asymmetry of the protein matrix is not accounted for in this continuum model. For the low-lying VEEs, the shifts are more or less independent of the employed model system and they are transferable to the other multichromophoric complexes as well.

**Table 9:** *Q*_*y*_ Excitation for Different Chla Monomers and Dimers Calculated Using TD-DFT@CAMY-B3LYP/TZP with and without Implicit Solvation^a^

				*P*_D1_–*P*_D2_			
	exp.	M1	M2	M1 monomers		M2 monomers	
solv.	1.82	1.81	1.84	1.78	1.81	1.80	1.84
no solv.	1.94	1.98	1.99	1.93	1.95	1.94	1.96
diff.	0.12	0.17	0.15	0.15	0.14	0.14	0.12
Chl_D1_–P_D1_–P_D2_–Chl_D2_ (M1 Monomers)							
solv.		1.76	1.78	1.81	1.84		
no solv.		1.90	1.92	1.95	2.00		
diff.		0.14	0.14	0.14	0.16		

a All values are in eV.

## Appendix B

### Calculating the BSE Hamiltonian

The most time-consuming step in the solution of the BSE is to build the matrix elements of the 2-particle Hamiltonian, eq 20. Let us denote with the matrix **K**^(±)^, a column of **A** ± **B** as defined in (20), in the primary basis.

Within the density fitting method, we expand products of atomic orbitals in a basis of auxiliary functions. To introduce the PADF variant of this technique, we label atomic orbitals as μ, ν, κ, and λ, auxiliary functions as α, β, γ, and δ, and atomic centers as *A*, *B*, *C*.... We also define the convention that μ, α ∈ *A*, ν, β ∈ *B*, κ, γ ∈ *C*, and λ, δ ∈ *D*, that is, μ and α are only labeling functions centered on atom *A*, and so on. The PADF expansion of the products of AOs can then be written as

21

where the factor of 1/2 in the case *A* = *B* is introduced to facilitate evaluation with the same algorithm while avoiding double counting. Let us write (20), in the primary basis as

22

where the *b*_κλ_ are elements of the transition density matrix and the *K*_μν_^(±)^ denote the matrix elements of a column of (**A** ± **B**)^(*n*+1)^. Inserting (21), the contribution to **K**^(±)^ for all atom pairs (*A*, *B*) is

23

where

24

In these and in the following quantities, the matrices are restricted to the primary basis functions centered on the atoms denoted by the indices in the superscripts. We define the intermediates

25

and

26

Here, *W*(ω = 0)_βγ_ are the matrix elements of the statically screened interaction in the basis of the auxiliary functions

27

We can then write

28

where in the + case *b* is symmetric and antisymmetric otherwise. These are the working equations with which (20) is implemented. They are similar to the ones for the self-energy, outlined in ref (148).

## Appendix C

### Elimination of Diffuse Functions from the Primary Basis

In addition to the usual canonical orthonormalization^185^ during the SCF prior to the qsGW calculations we herein introduce an additional step in order to improve the numerical stability of our algorithm. To project out too diffuse functions from the primary basis, we first diagonalize the overlap matrix of primary basis functions **S**

29

We then remove a column *u*_*i*_ from the transformation matrix if the corresponding eigenvalue λ_*i*_ is smaller than some predefined threshold ϵ_*s*_. We then define

30

and use this projector to transform all matrices in the primary basis, the Green’s functions, the self-energy contributions, as well as the matrices defined in (20) according to

31

where **K**′ would be the original exchange-like matrix in the primary basis including the diffuse part. This transformation is not necessary if a very large auxiliary basis set is used and is switched off in that case.

## Supporting Information Available

All structures used in this work (ZIP)
VEEs of chlorophyll dimers for different optimized geometries and crystal structures; evGW single-particle energies of the hexameric complex; and dominant particle-hole contributions; lowest TD-DFT/ωB97-X/TZP, evGW@PBEH40-BSE/TZP, and qsGW@-BSE/TZP excited states (PDF)
