# Supplementary material for: Quasiparticle Self-Consistent GW-Bethe–Salpeter Equation Calculations for Large Chromophoric Systems
Source: J Chem Theory Comput. 2022 Oct 6;18(11):6779–93. doi: 10.1021/acs.jctc.2c00531 (PMC9648197; doi:10.1021/acs.jctc.2c00531)
Supplement: Supplementary file 2 — ct2c00531_si_002.pdf [file ct2c00531_si_002.pdf]

Supporting information to: Quasiparticle Self-Consistent GW-Bethe-Salpeter equation calculations for large chromophoric systems

Arno Förster* and Lucas Visscher

Theoretical Chemistry, Vrije Universiteit, De Boelelaan 1083, NL-1081 HV, Amsterdam, The Netherlands

E-mail: a.t.l.foerster@vu.nl

1 VEEs of Chlorophyll dimers for different optimized geometries

Table S1: The lowest six excitation energies of a Chla dimer (monomer geomtry of figure 2a in the main text) All values are in eV. The structures have been optimized in this work at CAM-B3LYP-D3(BJ)/TZP.

kernel	Ω_1	Ω_2	Ω_3	Ω_4	Ω_5	Ω_6
exp. (VEE)[1]	1.95 (estimated)					
exp. (band max)[1]	1.90					
Ma dimer (figure 2a in main text, 108 atoms)						
evGW@LDA	1.98	1.99	2.16	2.22	2.51	2.64
evGW@PBEH40	1.97	2.02	2.24	2.27	2.58	2.67
qsGW	1.94	1.98	2.25	2.28	2.56	2.68
CAMY-B3LYP	2.12	2.16	2.38	2.43	2.51	2.61
ωB97-X	2.05	2.10	2.63	2.68	3.10	3.27
M2 dimer (figure 2b in main text, 140 atoms)						
evGW@LDA	1.96	2.00	2.17	2.24	2.48	2.64
evGW@PBEH40	1.97	1.98	2.26	2.29	2.50	2.67
qsGW	1.94	1.96	2.25	2.28	2.51	2.68
CAMY-B3LYP	2.12	2.14	2.34	2.42	2.49	2.61
ωB97-X	2.06	2.08	2.65	2.67	3.04	3.27
M3 dimer (figure 2b in main text, 178 atoms)						
evGW@LDA	1.98	2.02	2.08	2.11	2.31	2.42
evGW@PBEH40	1.96	1.98	2.13	2.15	2.33	2.43
qsGW	1.95	1.97	2.14	2.16	2.35	2.43
CAMY-B3LYP	2.15	2.18	2.25	2.32	2.38	2.43
ωB97-X	2.10	2.11	2.57	2.61	2.84	2.90

Table S2: The lowest 2 excitations of the Chlorophyll dimer (M3 structure in figure 2b in the main text) optimized at different geometries calculated with different methods.

	CAM-B3LYP-D3(BJ)				B3LYP-D3(BJ)		PBE-D4		PBE	
	TZP		TZ3P				TZP			
qsGW	1.94	1.98	1.92	1.96	1.82	1.88	1.83	1.85	1.84	1.86
evGW@LDA	1.98	1.99	1.98	1.99			1.86	1.88		
evGW@PBEH40	1.97	2.02	2.00	2.04			1.86	1.88		
CAMY-B3LYP[a]									2.03	2.08
CAMY-B3LYP[b]	2.13	2.16			1.96	2.04	2.01	2.02	2.01	2.05
ωB97-X	2.05	2.10								

2 VEEs of Chlorophyll dimers for different crystal structures

Table S3: Comparison of the Q_y excitation energies obtained with different methods and experimental values. The geometries are based on chrystal structures. All values are in eV.

	D140		D164	
evGW@LDA	1.78	1.81	1.78	1.86
evGW@PBEH40	1.71	1.75	1.73	1.77
qsGW	1.71	1.74	1.74	1.77
CAMY-B3LYP	1.93	1.95	1.94	1.96
exp. (VEE)[1]	1.95 (estimated)			
exp. (band max)[1]	1.90			

In contrast to the GW-BSE VEEs, the CAMY-B3LYP-TD-DFT results for the crystal structures are in excellent agreement with the available experimental gas-phase data.[2–4] In light of the factors just discussed, the excellent agreement of the CAMY-B3LYP-TD-DFT calculations is most likely due to an overestimation of the true VEEs (compares to the results shown in the main text and in table S2) which then cancels with the errors due to inadequate geometries.

3 evGW single-particle energies of the hexameric complex

The $evGW$@PBEH40 single-particle energies for the hexameric complex shown in table S4 do not change their order compared to the KS-DFT single-particle energies.

Table S4: The five highest occupied and the five lowest unoccpied single-particle energies at the KS-DFT (PBEH40) and the evGW@PBEH40 level of theory. The difference between the energy levels is shown in the last column.

index	E(KS) [eV]	E(evGW) [eV]	$\Delta_{KS-evGW}$
		occupied	
932	-6.759	-6.911	0.152
933	-6.716	-6.794	0.078
934	-6.674	-6.763	0.089
935	-6.626	-6.650	0.024
936	-6.595	-6.624	0.028
		virtual	
937	-3.601	-2.453	-1.148
938	-3.543	-2.376	-1.167
939	-3.517	-2.418	-1.099
940	-3.514	-2.327	-1.186
941	-3.511	-2.334	-1.177

3.1 TD-DFT/ωB97-X/TZP

Table S5: The lowest TD-DFT/ωB97-X/TZP excited states of the hexameric chromophore complex in the RC of PSII.[a].

	VEE	f	Character	weight
Ω_1	1.92	0.33	$\text{Chl}_{\text{D2}}{}^*$	0.47
Ω_2	1.93	0.64	$\text{Pd}_{\text{D2}}{}^*$	0.23
			$\text{Pd}_{\text{D1}}{}^*$	0.14
			$\text{Pd}_{\text{D1}}{}^+$ - $\text{Pd}_{\text{D2}}{}^-$	0.14
			$\text{Pd}_{\text{D2}}{}^+$ - $\text{Pd}_{\text{D1}}{}^-$	0.12
Ω_3	1.94	0.14	$\text{Pd}_{\text{D1}}{}^*$	0.23
			$\text{Chl}_{\text{D1}}{}^*/\text{Chl}_{\text{D1}}{}^+$ - $\text{Pheo}_{\text{D1}}{}^-$	0.18
			$\text{Chl}_{\text{D2}}{}^*$	0.09
			$\text{Chl}_{\text{D1}}{}^*/\text{Chl}_{\text{D1}}{}^+$ - $\text{Pheo}_{\text{D1}}{}^-$	0.09
Ω_4	1.96	0.18	$\text{Pheo}_{\text{D1}}{}^*/\text{Pheo}_{\text{D1}}{}^+$ - $\text{Chl}_{\text{D1}}{}^-$	0.16
			$\text{Pheo}_{\text{D1}}{}^*/\text{Pheo}_{\text{D1}}{}^+$ - $\text{Chl}_{\text{D1}}{}^-$	0.14
			$\text{Pheo}_{\text{D2}}{}^*$	0.13
			$\text{Pd}_{\text{D2}}{}^*$	0.09
			$\text{Pd}_{\text{D1}}{}^*$	0.09
Ω_5	1.97	0.09	$\text{Pheo}_{\text{D2}}{}^*$	0.34
			$\text{Chl}_{\text{D2}}{}^*$	0.11
Ω_6	1.98	0.07	$\text{Chl}_{\text{D1}}{}^*$	0.22
			$\text{Chl}_{\text{D1}}{}^*/\text{Chl}_{\text{D1}}{}^+$ - $\text{Pheo}_{\text{D1}}{}^-$	0.17
			$\text{Pheo}_{\text{D1}}{}^*/\text{Pheo}_{\text{D1}}{}^+$ - $\text{Chl}_{\text{D1}}{}^-$	0.10
			$\text{Pheo}_{\text{D1}}{}^*$	0.07

[a]Shown are the excitation energies Ω_S (in eV), the dominant coefficients of the corresponding eigenvector and the associated particle-hole transitions, as well as the oscillator strengths f.

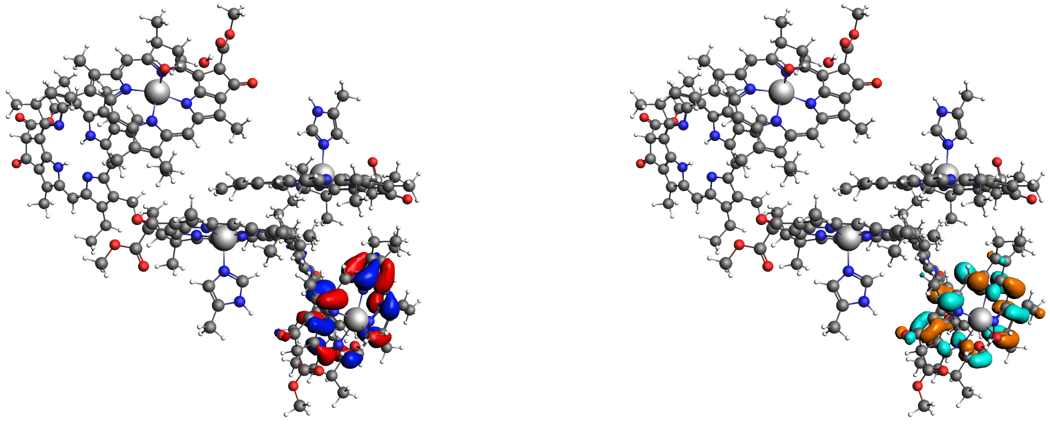

(a) Occupied Orbitals (b) Virtual Orbitals

Figure S1: Dominant particle-hole contributions to the first excited state of the hexameric complex using TD-DFT/ωB97-X/TZP.

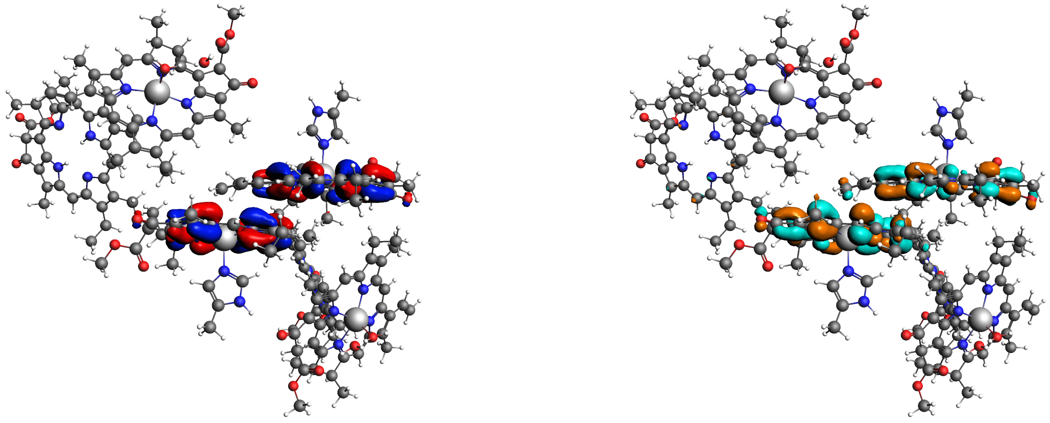

(a) Occupied Orbitals (b) Virtual Orbitals

Figure S2: Dominant particle-hole contributions to the second excited state of the hexameric complex using TD-DFT/ωB97-X/TZP.

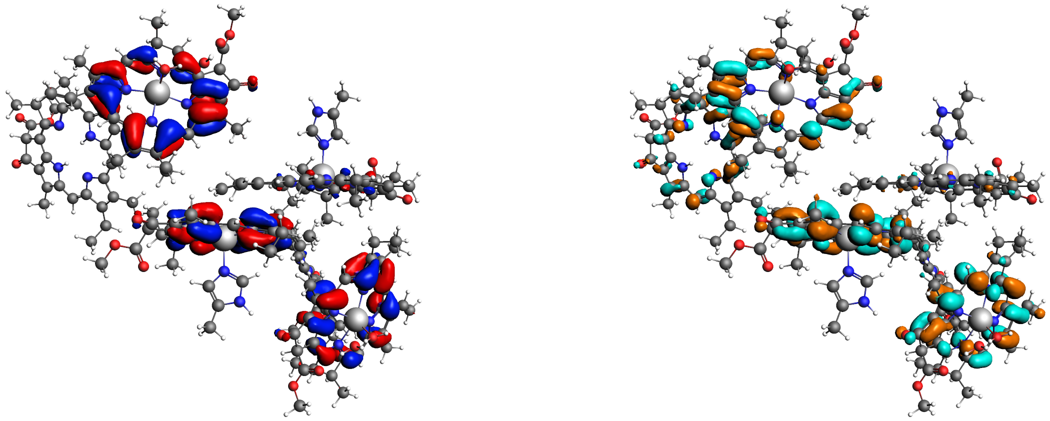

(a) Occupied Orbitals (b) Virtual Orbitals

Figure S3: Dominant particle-hole contributions to the 3rd excited state of the hexameric complex using TD-DFT/ωB97-X/TZP.

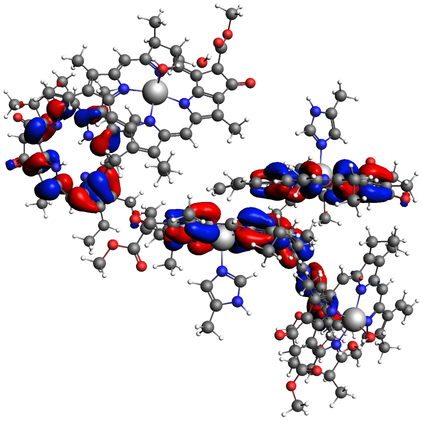

(a) Occupied Orbitals

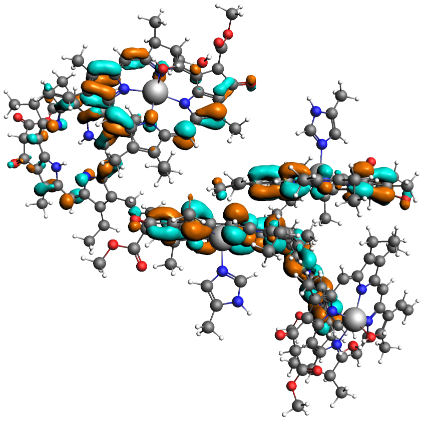

(b) Virtual Orbitals

Figure S4: Dominant particle-hole contributions to the 4th excited state of the hexameric complex using TD-DFT/ωB97-X/TZP.

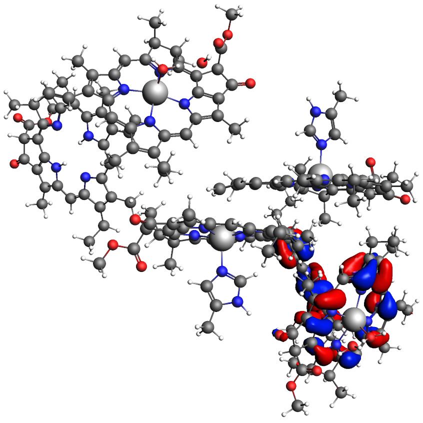

(a) Occupied Orbitals

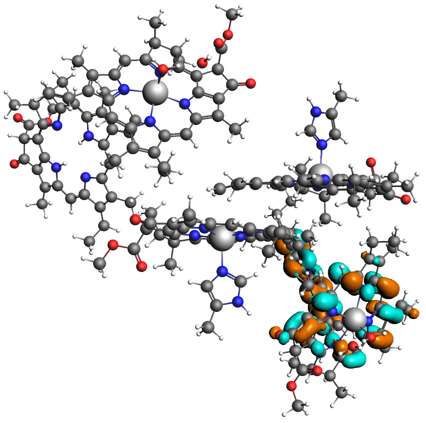

(b) Virtual Orbitals

Figure S5: Dominant particle-hole contributions to the 5th excited state of the hexameric complex using TD-DFT/ωB97-X/TZP.

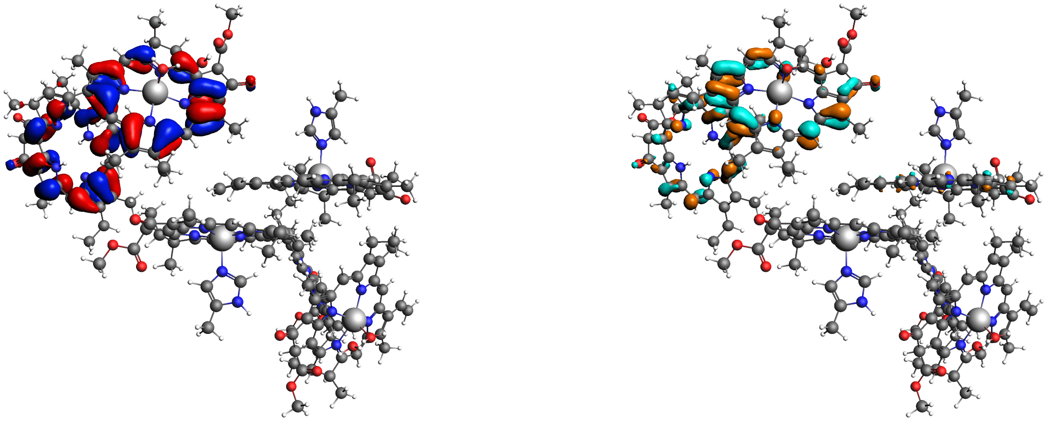

(a) Occupied Orbitals (b) Virtual Orbitals

Figure S6: Dominant particle-hole contributions to the 6th excited state of the hexameric complex using TD-DFT/ωB97-X/TZP.

3.2 evGW@PBEH40-BSE/TZP

Table S6: The lowest evGW@PBEH40-BSE/TZP excited states of the hexameric chromophore complex in the RC of PSII.[a].

	VEE	f	Character	weight
Ω_1	1.93	0.56	Chl_{D2}^*	0.33
			Pd_{D1}^*	0.32
Ω_2	1.94	0.48	Pd_{D2}^*	0.52
Ω_3	1.96	0.10	Pd_{D1}^*	0.28
			$Pheo_{D2}^*$	0.24
			Chl_{D2}^*	0.13
Ω_4	1.97	0.38	$Pheo_{D1}^*/Pheo_{D1}^+$ - Chl_{D1}^-	0.25
			$Pheo_{D1}^*/Pheo_{D1}^+$ - Chl_{D1}^-	0.17
			$Pheo_{D2}^*$	0.13
			Chl_{D1}^*	0.12
Ω_5	1.98	0.08	Chl_{D2}^*	0.26
			$Pheo_{D2}^*$	0.20
Ω_6	2.00	0.11	Chl_{D1}^*/Chl_{D1}^+ - $Pheo_{D1}^-$	0.36
			Chl_{D1}^*	0.22

[a]Shown are the excitation energies Ω_S (in eV), the dominant coefficients of the corresponding eigenvector and the associated particle-hole transitions, as well as the oscillator strengths f.

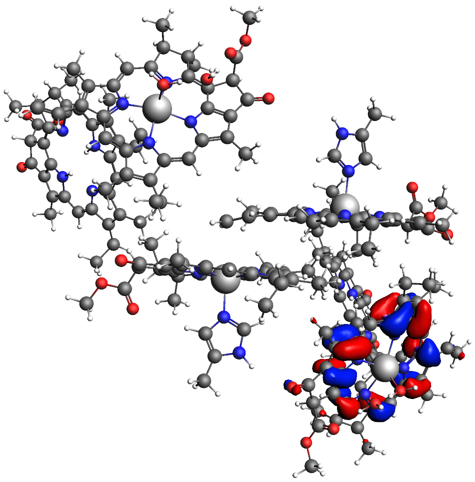

(a) Occupied Orbitals

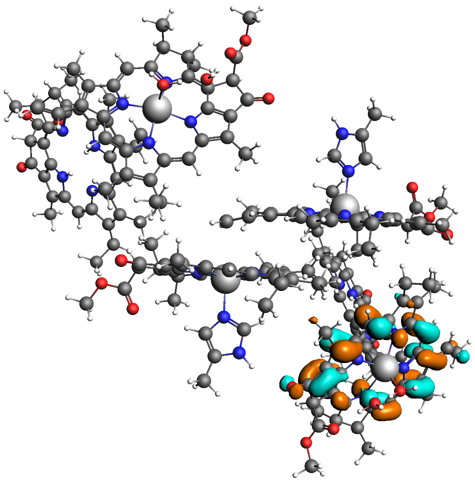

(b) Virtual Orbitals

Figure S7: Dominant particle-hole contributions to the first excited state of the hexameric complex using evGW@PBEH40-BSE/TZP.

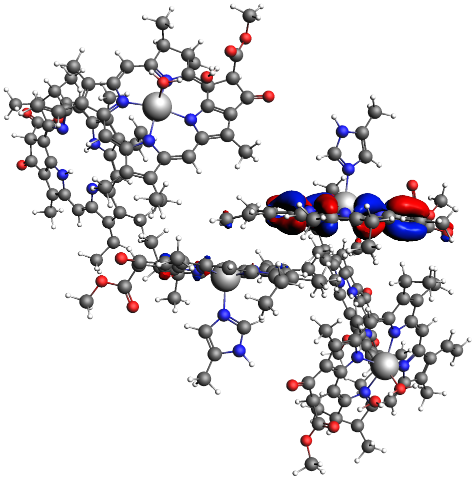

(a) Occupied Orbitals

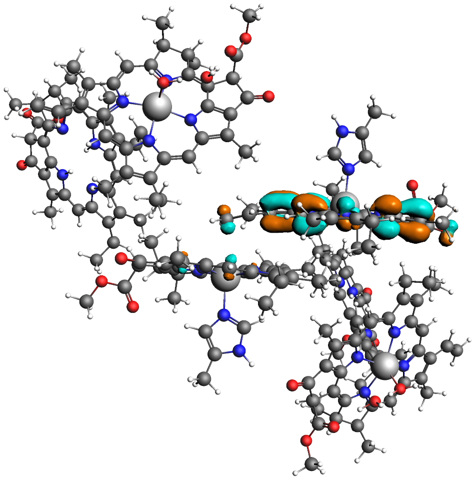

(b) Virtual Orbitals

Figure S8: Dominant particle-hole contributions to the second excited state of the hexameric complex using evGW@PBEH40-BSE/TZP.

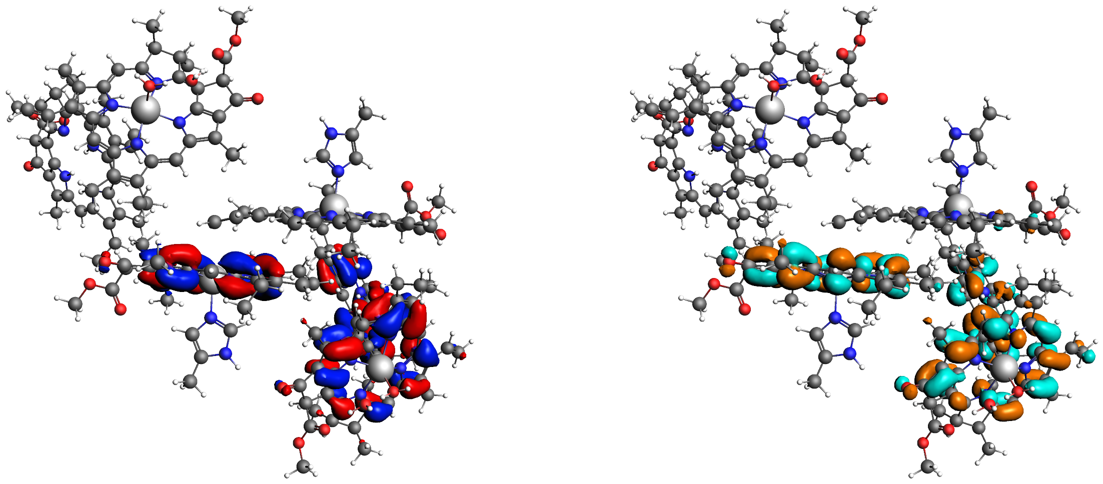

(a) Occupied Orbitals (b) Virtual Orbitals

Figure S9: Dominant particle-hole contributions to the 3rd excited state of the hexameric complex using evGW@PBEH40-BSE/TZP.

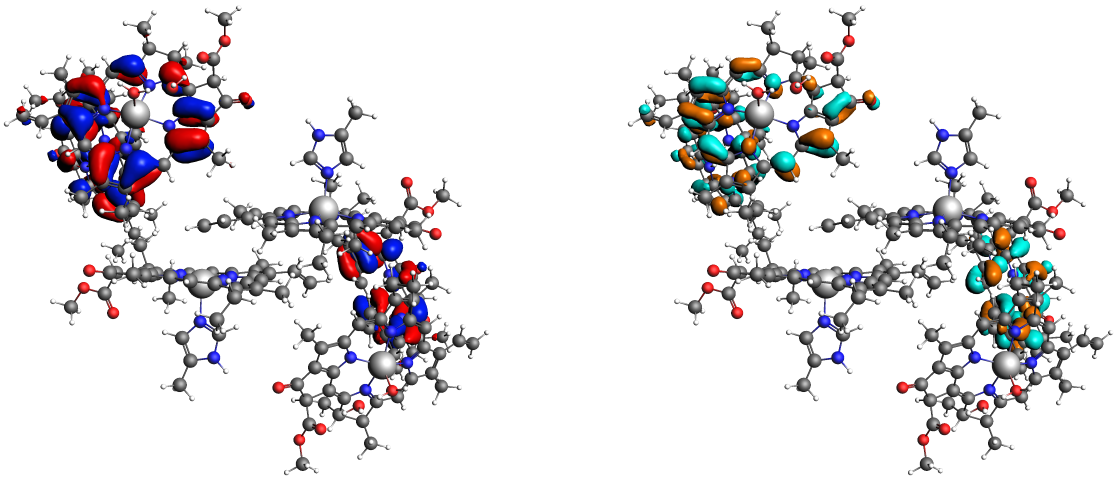

(a) Occupied Orbitals (b) Virtual Orbitals

Figure S10: Dominant particle-hole contributions to the 4th excited state of the hexameric complex using evGW@PBEH40-BSE/TZP.

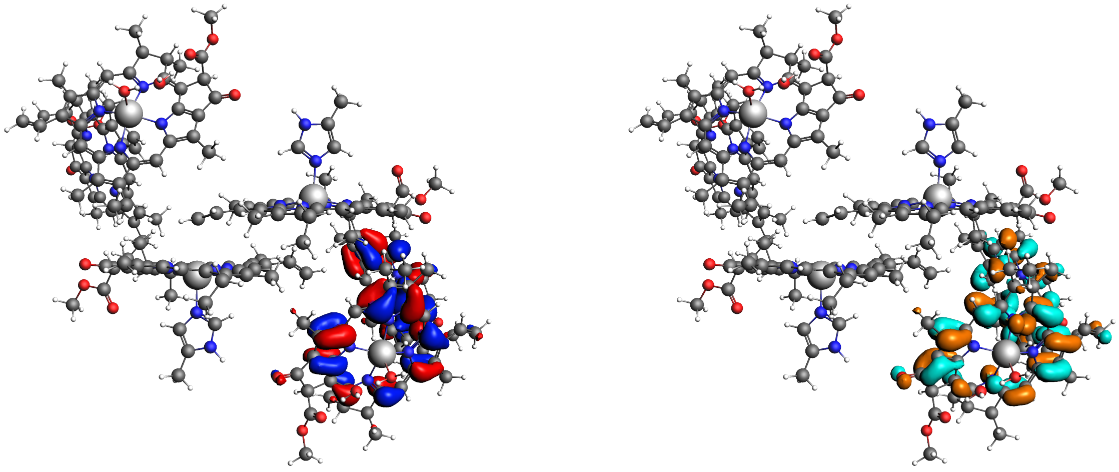

(a) Occupied Orbitals (b) Virtual Orbitals

Figure S11: Dominant particle-hole contributions to the 5th excited state of the hexameric complex using evGW@PBEH40-BSE/TZP.

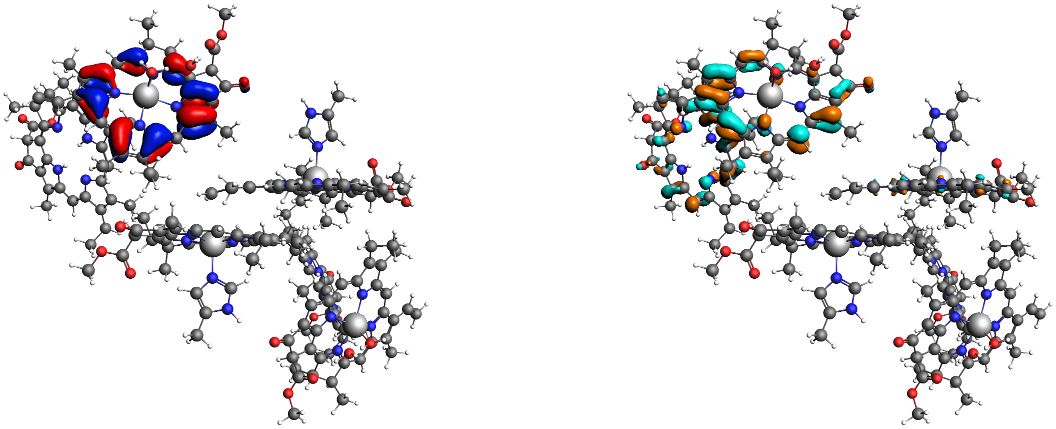

(a) Occupied Orbitals (b) Virtual Orbitals

Figure S12: Dominant particle-hole contributions to the 6th excited state of the hexameric complex using evGW@PBEH40-BSE/TZP.

3.3 qsGW@-BSE/TZP

Table S7: The lowest qsGW@-BSE/TZP excited states of the hexameric chromophore complex in the RC of PSII.[a].

	VEE	f	Character	weight
Ω_1	1.89	0.22	$\mathrm{Pd_{D2}}^*$	0.39
			$\mathrm{Chl_{D2}}^*$	0.22
Ω_3	1.90	0.77	$\mathrm{Pd_{D2}}^*$	0.24
			$\mathrm{Pd_{D1}}^*$	0.14
			$\mathrm{Pheo_{D2}}^*$	0.09
			$\mathrm{Pd_{D1}}^+$ - $\mathrm{Pd_{D2}}^-$	0.09
Ω_3	1.91	0.04	$\mathrm{Chl_{D1}}^*$	0.30
			$\mathrm{Pd_{D1}}^*$	0.24
			$\mathrm{Chl_{D1}}^+$ - $\mathrm{Pheo_{D1}}^-$	0.08
Ω_4	1.92	0.22	$\mathrm{Pheo_{D2}}^*$	0.39
			$\mathrm{Chl_{D2}}^*$	0.16
			$\mathrm{Pheo_{D2}}^*$	0.12
			$\mathrm{Chl_{D1}}^*$	0.09
Ω_5	1.94	0.01	$\mathrm{Chl_{D1}}^*$	0.23
			$\mathrm{Chl_{D2}}^*$	0.18
			$\mathrm{Pd_{D1}}^*$	0.16
			$\mathrm{Pd_{D2}}^*$	0.15
Ω_6	1.97	0.20	$\mathrm{Pheo_{D1}}^*$	0.54
			$\mathrm{Pheo_{D1}}^-$ - $\mathrm{Chl_{D1}}^+$	0.21
Ω_{13}	2.71	0.00	$\mathrm{Pd_{D2}}^+$ - $\mathrm{Chl_{D2}}^-$	0.81
			$\mathrm{Pd_{D1}}^+$ - $\mathrm{Chl_{D2}}^-$	0.13
Ω_{14}	2.73	0.00	$\mathrm{Pd_{D1}}^+$ - $\mathrm{Chl_{D1}}^-$	0.70
			$\mathrm{Pd_{D1}}^+$ - $\mathrm{Pheo_{D1}}^-$	0.20

[a]Shown are the excitation energies Ω_S (in eV), the dominant coefficients of the corresponding eigenvector and the associated particle-hole transitions, as well as the oscillator strengths f.

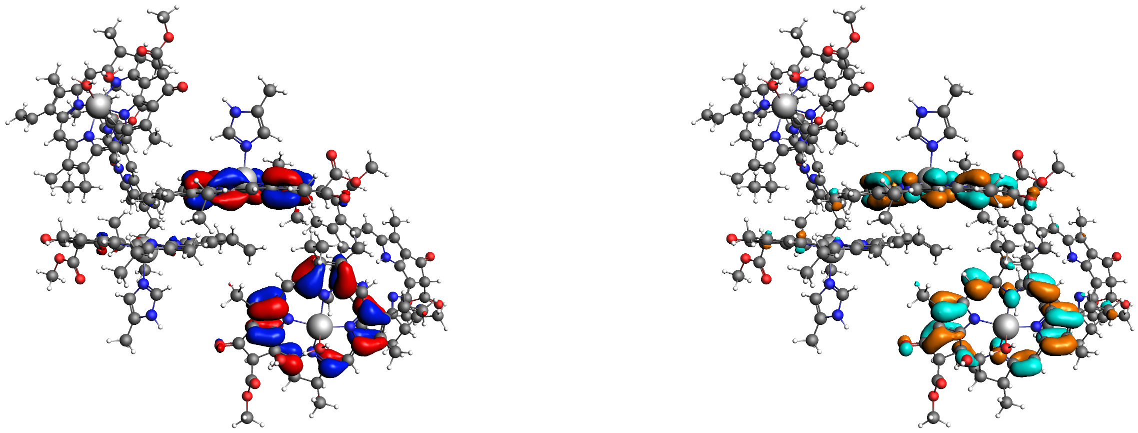

(a) Occupied Orbitals (b) Virtual Orbitals

Figure S13: Dominant particle-hole contributions to the first excited state of the hexameric complex using qsGW@-BSE/TZP.

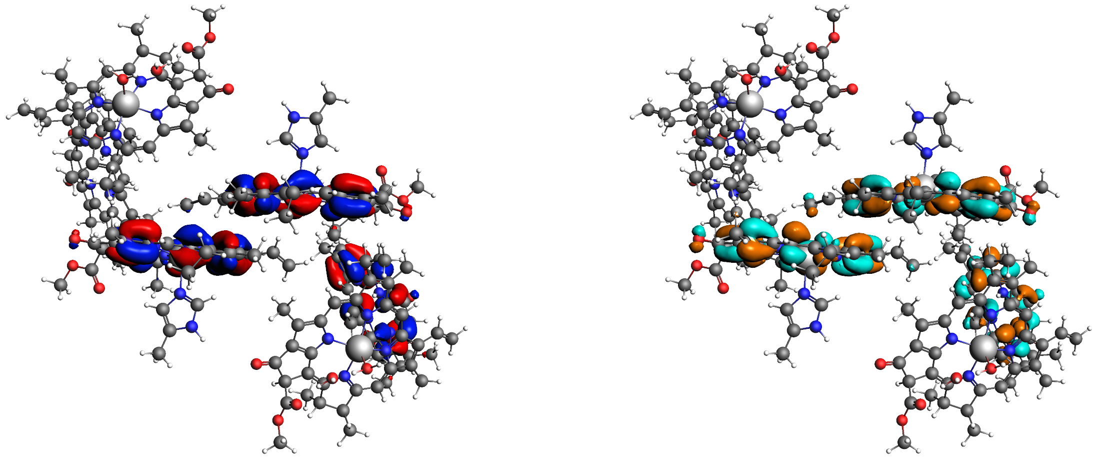

(a) Occupied Orbitals (b) Virtual Orbitals

Figure S14: Dominant particle-hole contributions to the second excited state of the hexameric complex using qsGW@-BSE/TZP.

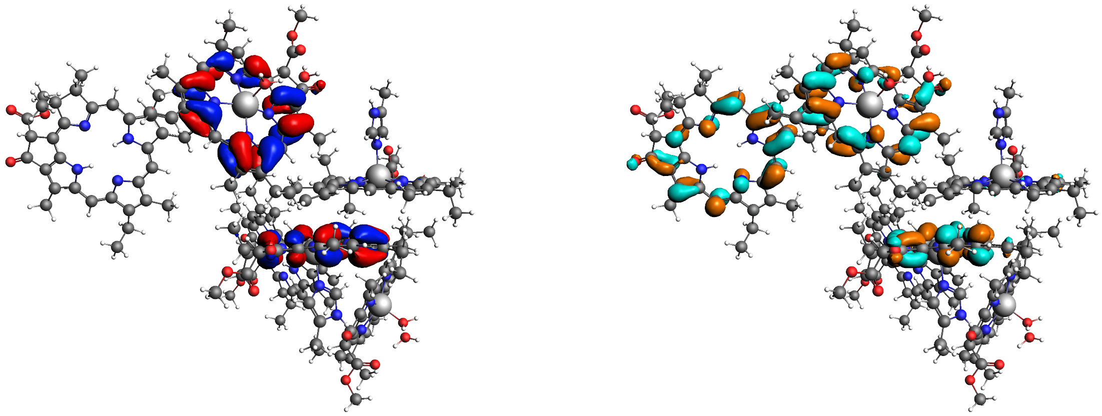

(a) Occupied Orbitals

(b) Virtual Orbitals

Figure S15: Dominant particle-hole contributions to the 3rd excited state of the hexameric complex using qsGW@-BSE/TZP.

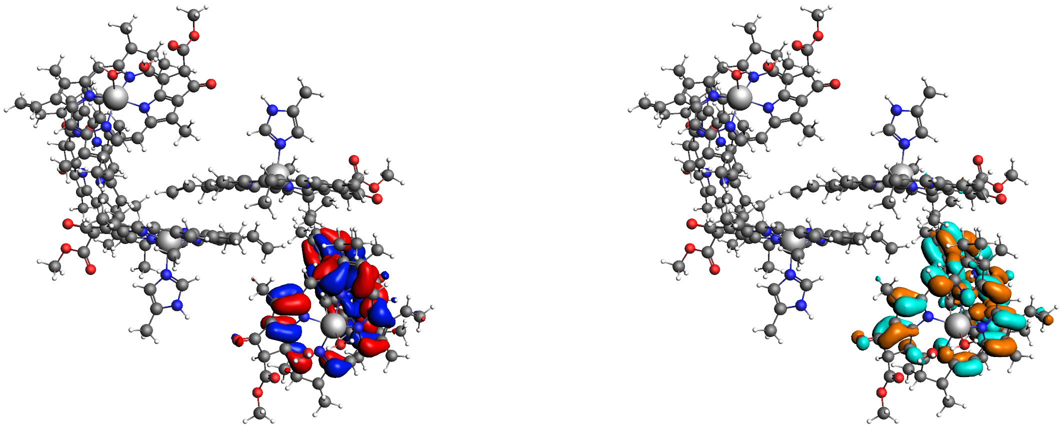

(a) Occupied Orbitals

(b) Virtual Orbitals

Figure S16: Dominant particle-hole contributions to the 4th excited state of the hexameric complex using qsGW@-BSE/TZP.

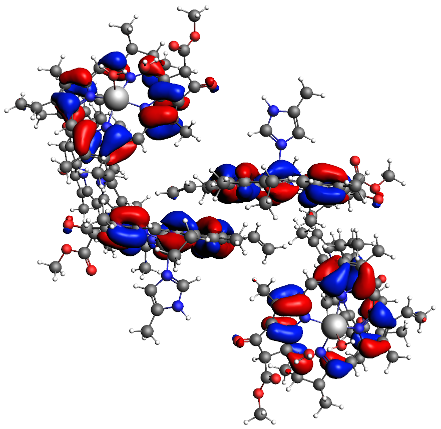

(a) Occupied Orbitals

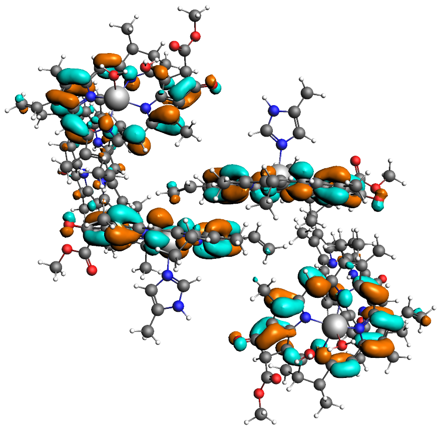

(b) Virtual Orbitals

Figure S17: Dominant particle-hole contributions to the 5th excited state of the hexameric complex using qsGW@-BSE/TZP.

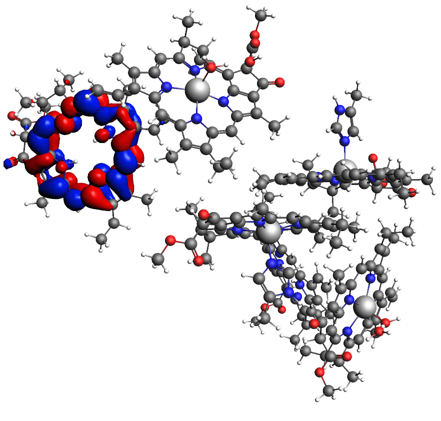

(a) Occupied Orbitals

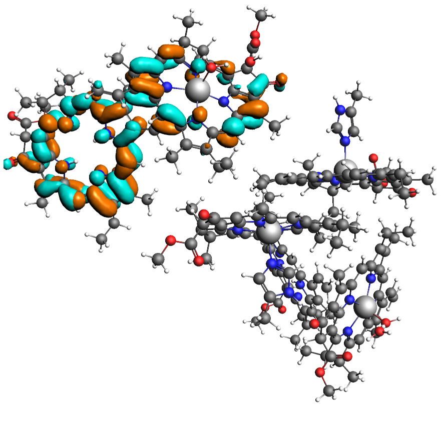

(b) Virtual Orbitals

Figure S18: Dominant particle-hole contributions to the 6th excited state of the hexameric complex using qsGW@-BSE/TZP.

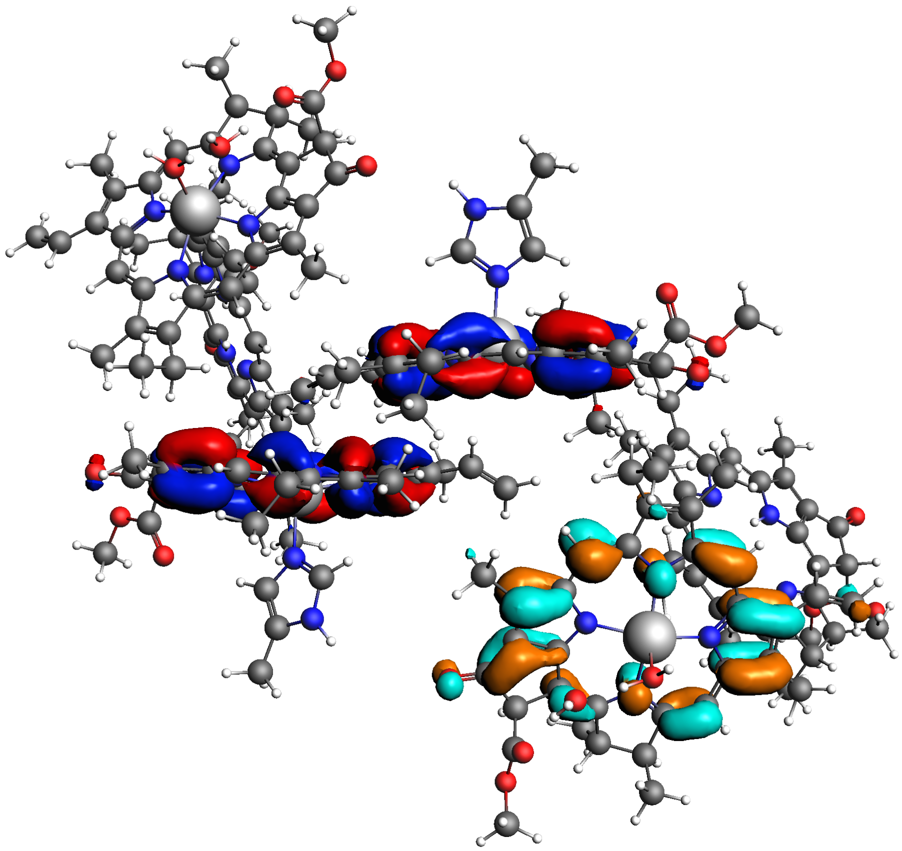

Figure S19: First excited state (Ω_{13}) of the hexameric complex with pronounced CT character using qsGW@-BSE/TZP.

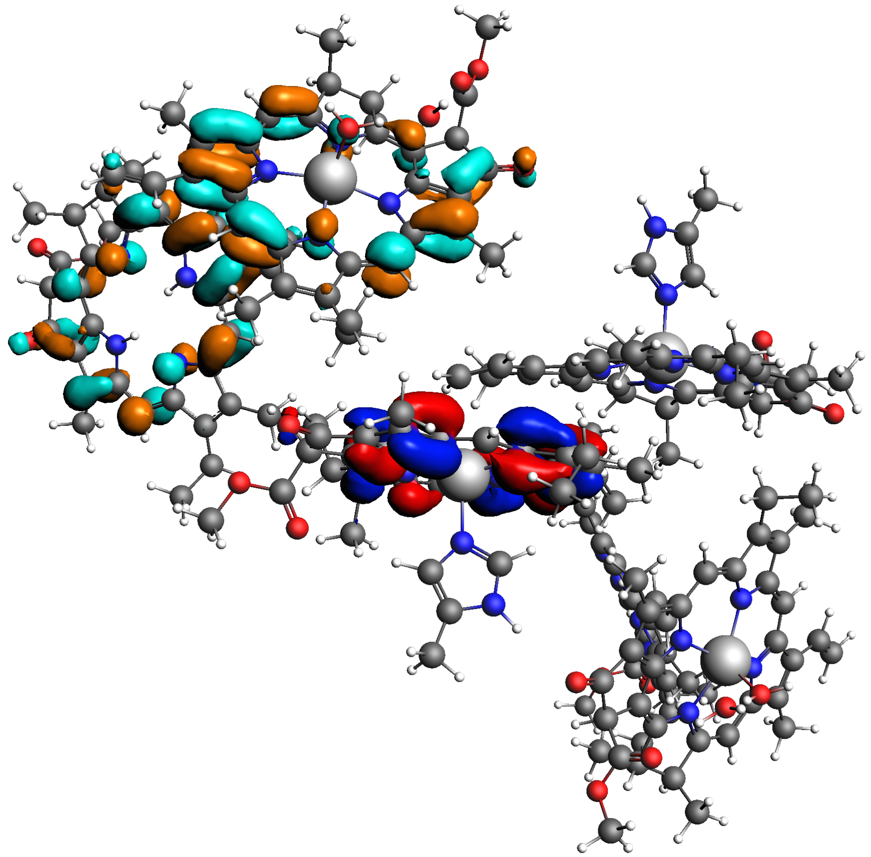

Figure S20: Second excited state ($\Omega_1 4$) of the hexameric complex with pronounced CT character using qsGW@-BSE/TZP.

References

(1) Milne, B. F.; Kjær, C.; Houmøller, J.; Stockett, M. H.; Toker, Y.; Rubio, A.; Nielsen, S. B. On the exciton coupling between two chlorophyll pigments in the absence of a protein environment: Intrinsic effects revealed by theory and experiment. *Angew. Chemie - Int. Ed.* **2016**, *55*, 6248–6251.

(2) Milne, B. F.; Toker, Y.; Rubio, A.; Nielsen, S. B. Unraveling the intrinsic color of chlorophyll. *Angew. Chemie - Int. Ed.* **2015**, *54*, 2170–2173.

(3) Gruber, E.; Kjær, C.; Nielsen, S. B.; Andersen, L. H. Intrinsic Photophysics of Light-harvesting Charge-tagged Chlorophyll a and b Pigments. *Chem. - A Eur. J.* **2019**, *25*, 9153–9158.

(4) Sirohiwal, A.; Neese, F.; Pantazis, D. A. How Can We Predict Accurate Electrochromic Shifts for Biochromophores? A Case Study on the Photosynthetic Reaction Center. *J. Chem. Theory Comput.* **2021**, *17*, 1858–1873.
